# Canada’s highest court unchains injection drug users; implications for harm reduction as standard of healthcare

**DOI:** 10.1186/1477-7517-9-34

**Published:** 2012-07-20

**Authors:** Dan Small

**Affiliations:** 1 PHS Community Services Society, 20 West Hastings Street, Vancouver, British Columbia, V6B 1G6, Canada; 2 Department of Anthropology, University of British Columbia, 6303 NW Marine Drive, Vancouver, British Columbia, V6T 1Z1, Canada

## Abstract

North America’s only supervised injection facility, Insite, opened its doors in September of 2003 with a federal exemption as a three-year scientific study. The results of the study, evaluated by an independent research team, showed it to be successful in engaging the target group in healthcare, preventing overdose death and HIV infections while increasing uptake and retention in detox and treatment. The research, published in peer-reviewed medical and scientific journals, also showed that the program did not increase public disorder, crime or drug use. Despite the substantial evidence showing the effectiveness of the program, the future of Insite came under threat with the election of a conservative federal government in 2006. As a result, the PHS Community Services Society (PHS), the non-profit organization that operates Insite, launched a legal case to protect the program. On 30 September 2011, Supreme Court of Canada ruled in favour of Insite and underscored the rights of people with addictions to the security of their person under section 7 of the Charter of Rights and Freedoms (*Charter of Rights*). The decision clears the ground for other jurisdictions in Canada, and perhaps North America, to implement supervised injection and harm reduction where it is epidemiologically indicated. The legal case validates the personhood of people with addictions while metaphorically unchaining them from the criminal justice system.

## 

“The philosophers have only interpreted the world in various ways; the point, however, is to *change* it” [1].

After a long legal and cultural battle, North America’s only supervised injection facility, Insite, is finally safe from arbitrary political interference. This was a case where personal experience, activism, advocacy, medicine and science stood side-by-side to protect the rights of even the most marginalized members of the community to life-saving healthcare. The case began at the Supreme Court of British Columbia and eventually made its way to the Supreme Court of Canada (SCC). At the centre of the case were the personal stories of people who relied on supervised injection to stay alive together with testimony from scientists, physicians, healthcare officials and the operators of Insite. On 30 September 2011, Supreme Court of Canada drew a legal line in the sand that highlights the rights of people with addictions to the security of their person under section 7 of the Charter of Rights and Freedoms (*Charter of Rights*) [2]. The Charter enshrines the values of Canadian culture regarding the rights of individuals with respect to the provincial, federal and territorial governments. The judges of Canada’s highest court are appointed from a wide variety of political backgrounds. The decision was unanimous and reinforced the foundation of our understanding of addiction as a healthcare matter in Canada.

As a caveat, I am not a distant academic examining supervised injection from the point of view of a removed observer. I am part of the senior management of the non-profit organization that founded and operates Insite, the PHS Community Services Society (PHS) and, as such, I have been intricately involved in the development, set-up, management and advocacy for Insite. I am a participant observer and so this commentary is written from the point of view of my personal experience.

The journey for Insite has been wrought with challenges because it confronts our inner web of belief about how to best approach addiction. Supervised injection exists in a moral minefield at the very heart of our culture. By culture, I am speaking about what we believe to be right and wrong, the implicit and explicit values that are the building blocks of our understanding, practice and societal approaches to people with addictions [3]. As such, it is my belief that this legal victory is about something much more fundamental than the legal, medical or scientific issues that arise from it. It points the way towards a shift in our cultural orientation that allows for addiction to be constructed as a social issue best addressed, metaphorically, by the Chief of Medicine rather than the Chief of Police (Figure 1).

**Figure 1:**
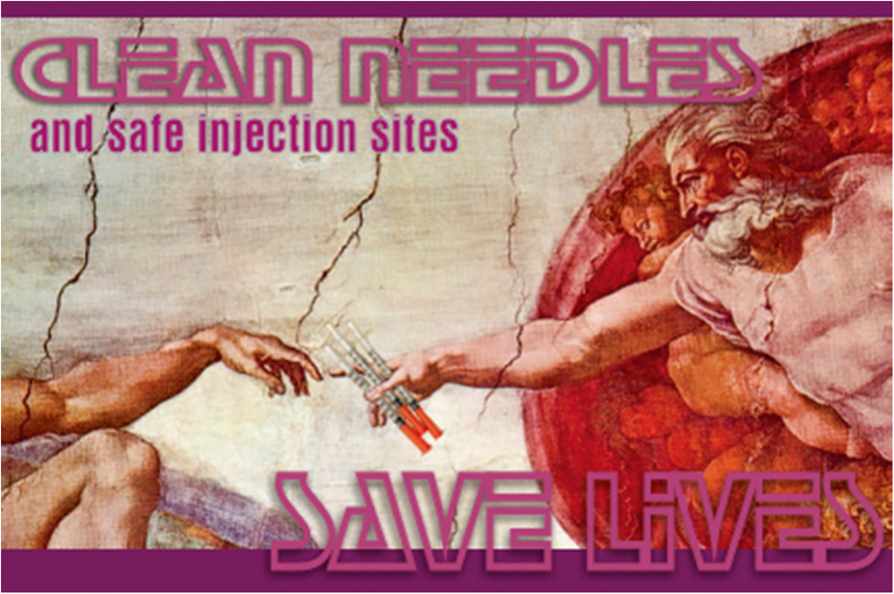
**God distributing clean needles.** Graphic by Flux Design.

This cultural change is best illustrated by a story from only a decade ago when harm reduction was not widely accepted or understood in Canada. Harm reduction innovations involve attempting to reduce harms associated with drugs, such as fatal overdoses, but to do so without necessary eliminating the use of drugs (abstinence). The Harm Reduction Journal provides the following definition:

“We define 'harm reduction' as 'policies and programs which aim to reduce the health, social, and economic costs of legal and illegal psychoactive drug use *without necessarily reducing* drug consumption'” [4].

In the year preceding the opening of Insite in 2003, I was collaborating on a draft press release in response to local opposition to harm reduction. The press release simply stated that addiction is, primarily, a healthcare matter. At that time, the notion of publicly stating that addiction is a healthcare rather than criminal justice issue was so controversial that I could not convince anyone in the establishment to lend their name to the press release. In the end, two community activists agreed to sign their names to what was, at the time, a provocative public statement. Now, ten years later; this has become an established legal fact in Canada. Addiction is a healthcare matter.

The PHS initiated the case at the Supreme Court of British Columbia in 2008 to protect the program from closure by a conservative federal government. We did so at a time when there was no formal institutional support for a struggle to protect Insite in the courts. The only intervener was the British Columbia Civil Liberties Association. Despite their role in co-managing Insite, Vancouver Coastal Health (VCH) counseled our organization not to turn to the courts to protect the program and refused to provide any formal assistance for the PHS to legally defend it. In the initial case, neither Vancouver Health Authority (VCH), nor the Attorney General of British Columbia sought intervener status. Fortunately, the PHS was able to obtain pro bono representation by three lawyers: Monique Pongrecic-Speier, F. Andrew (Drew) Schroeder and Joseph Arvay all of whom nobly took on the case when it felt like Insite had been backed against the wall. A related and important case, entered by the Vancouver Area Network of Drug Users (Respondent/Appellant on cross appeal) was heard jointly.

The PHS case focused on two overarching themes. The first theme related to the division of powers between the federal and provincial governments and essentially argued that the operation of Insite falls under the jurisdiction of the Province of British Columbia. The second argument pertained to the Charter. The Charter is the first part of the Constitution of Canada and contains a passage of particular relevance to Insite.^2^ This portion of the Charter, section 7, is of central importance to Insite and states that: “Everyone has the right to life, liberty and security of the person and the right not to be deprived thereof except in accordance with the principles of fundamental justice” [5](p. 4). The PHS argued the federal Health Minister’s withholding of an exemption from the Controlled Drugs and Substances Act (CDSA) that was required at that time for Insite’s operation was prejudicial and arbitrary. More so, it jeopardized the life chances of people who need the facility to access life saving healthcare.

The case at the BC Supreme Court laid the groundwork for the facts that would later form the foundation for the landmark ruling at the Supreme Court of Canada. Given the case’s importance, it is worth examining at a high level. There are, in my view, four key findings of fact in this first case.

The first key fact pertains to the notion that addiction is a healthcare matter. The Government of Canada conceded this as an indisputable fact.

The presiding judge at the BC Supreme Court, Justice Ian Pitfield, highlighted this absolutely critical cultural admission in his Reasons for Judgment when he stated: “drug addiction is an illness” [6] (p. 20). The declaration of addiction as an illness allows for the devotion of healthcare resources to addressing it.

The second fact that was established was that drugs, in and of themselves, do not necessarily cause serious interruptions in health. Rather, it is the method, mechanism and context within which drugs are ingested that brings about danger:

“Controlled substances such as heroin or cocaine that are introduced into the bloodstream by injection do not cause Hepatitis C or HIV/AIDS. Rather, the use of unsanitary equipment, techniques and procedures for injection permits the transmission of those infections, illnesses or diseases from one individual to another” [6] (p. 33, para. 87).

This is the foundation of supervised injection as an intervention. The point of intervention focuses on reducing the harms associated with drug use without forcing abstinence as a precondition for receiving healthcare.

The idea that drugs, as substances, are not automatically intrinsically evil or dangerous has been a culturally controversial notion. There are, broadly speaking, two competing overarching narratives about addiction [3].The first narrative focuses, essentially, on drug use and the drugs themselves as intrinsically dangerous. The second does not centre on the drugs themselves, but instead concentrates on the way drugs are administered (e.g. clean versus unclean syringes) and the psycho-socio-cultural context of their use (such as criminalization, poverty and mental pain).

This distinction can be traced to the work of psychologist Bruce K. Alexander who first discussed two orientations for constructing addiction 30 years ago [7]. The first orientation essentially constructs addiction as follows: first a person takes a drug, then, eventually, the drug takes the person as a result of repeated exposure. Understanding addiction in terms of an exposure to drug concentrates on the eradication of drugs as the point of intervention. Following on from this reasoning, it is the reduction in drug exposures that will ultimately reduce or eliminate addiction. The rival narrative about addiction, described in terms of coping or adapting by Alexander and his colleagues constructs the problem altogether differently. According to this alternative explanation, people take drugs; drugs do not take people. This perspective maintains that people misuse drugs due to impoverished conditions and psychosocial pain that require extraordinary coping strategies. In turning focus away from the dangers of the drugs themselves to the ways in which they are being used, the original court decision was aligned with this latter orientation.

The third legal fact pertained to the notion that effective medical interventions are available to measurably reduce the harms of addiction. The primary healthcare intervention in Insite is the provision of sterile injection equipment and the supervision of injection:

“The risk of morbidity and mortality associated with addiction and injection is ameliorated by injection in the presence of qualified health professionals” [6] (p. 33, para. 87).

The finding of this fact, based on the scientific and medical evidence before the court, established that a supervised injection facility helps to prevent disease and death. Contrary to its popular characterization as an isolated program, Insite also offers detox and treatment on site.

The final fact is perhaps the most culturally controversial because it foregrounds the fact that effective healthcare interventions exist for addiction that do not demand abstinence. Justice Pitfield understood that while Insite is not traditional treatment, it is, nonetheless, important healthcare:

“While users do not use Insite directly to treat addiction, they receive services and assistance at Insite which reduce the risk of overdose that is a feature of their illness, they avoid risk of being infected or of infecting others by injection and they gain access to counselling and consultation that may lead to abstinence and rehabilitation. All of this is healthcare” [6] (p. 51, para. 136).

This recognition of this fact feeds into the cultural anxieties about somehow enabling or encouraging addiction by not outlawing it with vehemence. The ruling also addressed this culturally notorious notion of overlooking addiction:

“Society cannot condone addiction, but in the face of its presence it cannot fail to manage it, hopefully with ultimate success reflected in the cure of the addicted individual and abstinence” [6] (p. 54., para. 144).

The rival perspective, abstinence at all cost, would presumably withhold supervised injection as a healthcare intervention even if it resulted in preventable fatal overdoses. This was the very reason the PHS entered the courts: we believe that harm reduction is a doorway into treatment, detox and abstinence and that the safeguarding of human life offered by supervised injection is sacrosanct. Without supervised injection, people might perish from fatal overdoses before realizing the opportunity to one day pursue detox, treatment and abstinence. Instead, all that would be left would be a mortality statistic: a faint reminder that they ever lived.

In summary, there were four legal facts that go to the heart of a particular cultural understanding of addiction:

1. Addiction is a healthcare matter.

2. Drugs to do not cause deadly HIV, HCV and fatal overdoses: unclean needles and unsupervised injection do.

3. Supervised injection is effective at preventing morbidity and mortality. Harm reduction opens the door to a range of healthcare (e.g. detox, treatment).

4. Abstinence, though laudable, is not compulsory for effective healthcare interventions, with measurable outcomes (e.g. such as saving lives by intervening in otherwise fatal overdoses or preventing HIV) for addiction. The idea of condoning or enabling addiction with supervised injection takes second place to keeping people alive.

The establishment of these four key facts, in my view, laid the groundwork for both a legal and a cultural victory with respect to the notion of supervised injection.

As the case advanced, it gathered cultural momentum as part of a growing acceptance of a particular understanding about addiction as described above. At the final stage, 14 interveners had joined the proceedings including: the Vancouver Coastal Health Authority, Canadian Nurses Association, British Columbia Nurses’ Union, Registered Nurses’ Association of Ontario, Association of Registered Nurses of British Columbia, Canadian Medical Association, Canadian Civil Liberties Association, Canadian HIV/AIDS Legal Network, International Harm Reduction Association, CACTUS Montreal (a non-profit organization dedicated to providing non-judgmental assistance and risk reduction for at risk individuals including those use illicit drugs, street involved youth, sex trade workers as well as transvestite and transsexual persons), Canadian Public Health Association, British Columbia Civil Liberties Association, Attorney General of Quebec and Dr. Peter AIDS Foundation. The Attorney General of British Columbia was a respondent with regard to the doctrine of inter-jurisdictional immunity that was won at the Appeal Court level and argued for localized control over Insite as a provincial initiative.

Sadly, there has always been a psychosocial phenomenon that is culturally expunged or relegated to the shadows due to the moral anxieties that it creates in the wider community. This has been true in the case in the present and past with issues or experiences that make people uncomfortable such as death, sexuality, mental illness or addiction which are sequestered in social life and institutional settings [8]. This process of sequestering, or hiding away of the social phenomena that alarm or anger us, also takes its shape in the form of cultural erasures and silences, things that are unsaid which can, in actuality, be more influential than what is said [9]. Addiction is just such a phenomenon; people with addictions have been sequestered, silenced and erased from positive social life. Their personhood has been so dramatically undermined that their identities are sometimes socially spoiled [10] leaving them metaphorically chained. Throughout the education campaign to save Insite, we attempted to combat this identity erosion by highlighting the personhood of people living with addictions by echoing the idea that everyone living with addictions was someone’s son or daughter (Figure 2).

**Figure 2:**
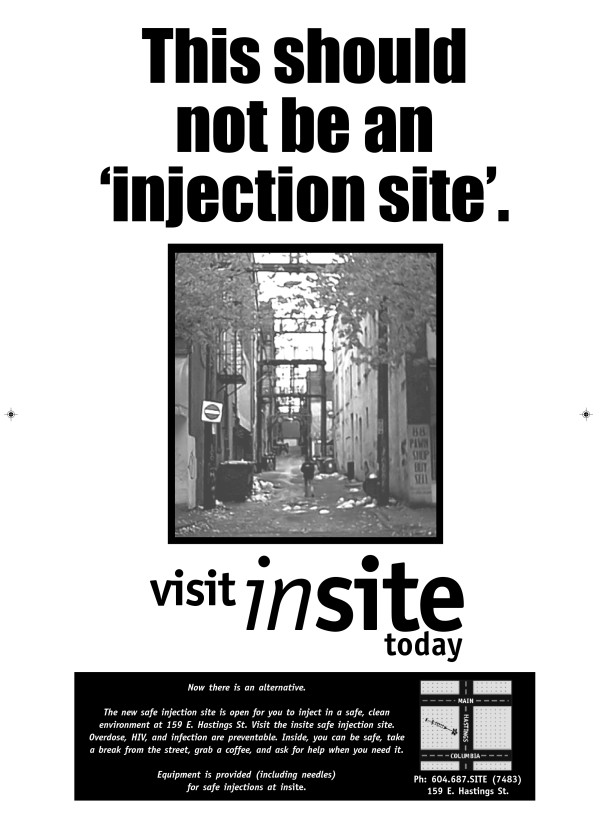
**This should not be an injection site.** Graphic by PHS Community Services Society.

The field of mental health provides an interesting analogy with respect to the process of liberation of people with pain from societal shackles of stigma and incarceration. Psychiatry’s approach to people with mental illnesses transitioned towards humanistic treatment in the 1780’s under the leadership of more humanistic psychiatrists and reforms in the mental health field. Physician Vincenzo Chiarugi (1759–1820) led a movement espousing the humane treatment of the mentally ill which took its first applied step, in practice, in 1788 with the opening of Hospital Bonifacio in Florence, Italy where he served as the physician director [11]. Chiarugi’s approach was in keeping with the goals of Grand Duke Pietro Leopold of Tuscany, a socially conscious aristocrat who ordered the establishment of Bonifazio, and was predicated on respectful and humane treatment [12]. He is a significant figure in the history of humanistic medicine and one of the fathers of compassionate psychiatry. Chiarugi is thought to be the first figure to forbid the use of chains to restrain the mentally ill (a policy which he established during his role as physician director of Santa Doretea hospital before 1793) [12].

Similarly, Jean-Baptiste Pussin (1746–1811) and Madame Marguerite Pussin (1754-?) helped to infuse compassion into the practice of mental health [12,13]. After having been a patient at Bicêtre hospital in the suburbs of Paris, Jean-Baptiste Pussin went on to become the director of a psychiatric ward from 1785 to 1802 during which time he implemented a series of compassionate improvements in the treatment of mentally ill [14]. Pussin outlawed the employment of chains to imprison the mentally ill in 1797 while serving as the governor of Bicêtre [12]. Pussin is an important forerunner in the history of humanistic mental health services.

In popular culture, physician Phillippe Pinel is widely thought of as being the first individual to liberate the mentally ill from chains. The renowned painting *Madwomen at the Salpêtrière*, painted by Tony-Robert Fleury, has helped to immortalize this legend. The painting shows Pussin removing the chains of psychiatric patients while Pinel looks on and symbolizes a transition towards more humanistic approaches to the mentally ill in the 18^th^ century. Today the painting hangs in the entrance to the Library Charcot in the Salpêtrière hospital [14].

In fact, it was Pussin who inspired Pinel to ban the use of chains for detaining people living with mental illnesses [12,15]. After having worked at Bicêtre between 1793 and 1795, Philippe Pinel was so inspired by the work of Pussin that he credited him with the emancipation of the mentally ill and the first actual application of humanistic psychiatric treatment. When Pinel moved to Salpêtrière, the largest hospital in Paris, he established a post for Pussin who took up the position there from 1801 until his death in 1811.14 At Salpêtrière in Paris, Pussin and Pinel worked together to apply humanistic approaches to psychiatric treatment.

There is some humanistic truth at the heart of legendary characterization of Pinel as the person that liberated the mentally ill from their chains. He did, in fact, showed significant leadership by moving away from abandonment and brutal imprisonment to a therapeutic approach based on medical science and compassion [12]. Similarly, the Insite legal case helps to make a similar transition from the cruelty of criminalization in addiction to a healthcare model where people with addiction have fundamental rights to life saving healthcare.

The mental health field transitioned from a model based on incarceration and neglect of the mentally ill to an approach based on compassion, science and medical treatment in the 18^th^ century. In the addiction field, this transition has taken another two centuries (21^st^ century). The metaphor of chaining of the addicted goes beyond the symbolic. In 2009, the US incarcerated more than 400,000 individuals for non-violent drug offenses (a greater number than those incarcerated for all offenses in the 27 nations of the European Union combined [16].

The incarceration of the addicted has been so dramatic that, when seen through the lens of epidemiology, it can be considered as a catastrophic event that has resulted in tremendous suffering and death. The epidemiological tool of years of life lost (YLL) is useful for examining the impact of a criminal justice approach to addiction. Drucker defines years of life lost as “the number of years between the victim’s age at death and the age that his or her usual life expectancy would predict. Thus, for the average American with a life expectancy of 75years, a child’s death at age ten implies a loss of sixty-five potential years of life [16] (p.69). Building on this logic, Drucker notes that 1,513 people died representing an estimated 47,000 YLL in the Titanic disaster, 2,819 deaths representing an estimated 104,303 YLL died in the World Trade Centre tragedy and reasons that over the past 35years (since the introduction of the Rockefeller drug laws in New York), more than 7 million people have been incarcerated. This translates into an estimated 14 million YLL representing 350,000 deaths in a group of the same age [16].

In 2009 alone, more than 400,000 individuals were incarcerated for non-violent drug offenses in the United States. This represents a greater number than all those incarcerated for all offenses in the 27 nations of the European Union combined [16]. The Insite victory is emblematic of a different cultural understanding of addiction that is supplanting a traditional one. This newer approach assumes people living with addictions are in need of healthcare rather than punishment through the criminal justice system.

By focusing on the federal Health Minister’s refusal to provide an exemption for Insite under the existing regulatory framework, the SCC did not have to make any alterations to existing provincial and federal jurisdictions over the program. Canada’s federal government attempted to argue that the federal minister of health had, technically, never “not given” a permit for Vancouver’s supervised injection site and therefore never formally jeopardized its operation. However, the court ruled that it was self-evident that the federal Health Minister had every intention to close Insite:

“The Minister of Health must be regarded as having made a decision whether to grant an exemption, since he considered the application before him and decided not to grant it. The Minister’s decision, but for the trial judge’s interim order, would have prevented injection drug users from accessing the health services offered by Insite, *threatening their health and indeed their lives* [emphasis added] [2] (p. 9).

The SCC concluded that the Minister’s intention to shut Insite would have threatened the lives of the people who rely on the program. It also noted the program would not have remained open had it not been for protection provided by the Supreme Court of BC (Figure 3).

**Figure 3:**
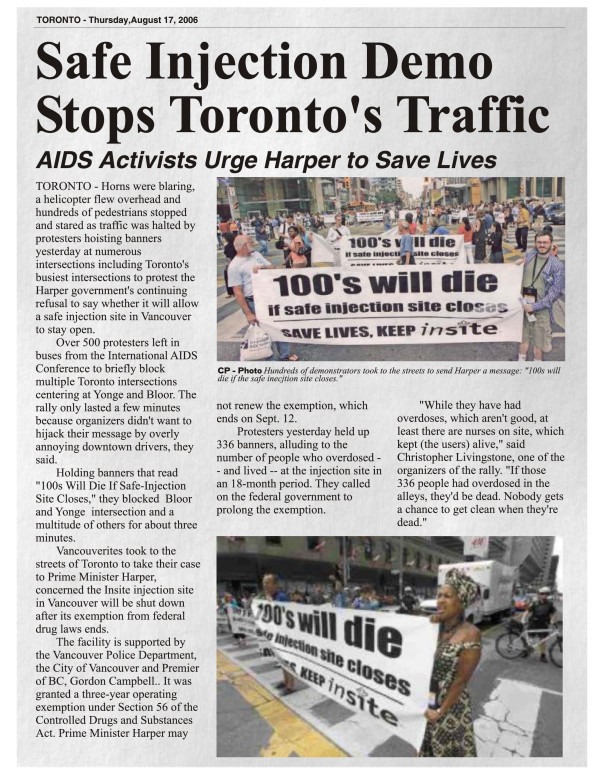
**Safe injection site demonstration.** Graphic by PHS Community Services Society.

National public policy with respect to supervised injection in Canada began with the establishment of a localized response to a healthcare emergency. Supervised injection, in this case, began from the ground up rather from a top down policy. The goals of Insite originated out of local need, inspired by the idea that people with addictions deserved something better than death from overdose. The goals and outcome measures were simple. The program aimed to provide a doorway to life, supported housing, physicians, healthcare services and supports. The intent of the program was to provide its interventions (e.g. clean syringes, supervised injection) in an accessible way without barriers (such as abstinence or onerous intake procedures).

One thing that is clearly demonstrated by the case of Insite is that science is not enough, on its own, to change public policy especially in stigmatized areas like addiction. The simple existence of a scientific evidence base does not automatically lead to changes in policies or practice. Policy makers and elected officials need to pay attention to the evidence base. In some disappointing instances, as the case of Insite has shown, policy makers need to be forced to pay attention to the established facts. Conversely, scientists need to take an active role in affecting public policy when the evidence indicates the need for change [17]. The SIF, for instance, had more than three-dozen peer-reviewed papers associated with its evaluation [18-47]. Despite the fact that much of the evaluation was paid for by the Government of Canada, they chose to ignore the scientific findings. The evidence base generated by Canada’s supervised injection trial should have earned it a medical exemption (the next stage of operational permit after the scientific authorization originally granted to Insite) but it was not provided. Uncompromising advocacy, including public protest and legal challenge, was required to obtain the permit.

In this way, science in healthcare and applied research are not the same [48]. While science in healthcare is portrayed as "objective", applied research is seen as rooted in the context of the community needs. From my perspective, science in healthcare needs to move more towards clinical application. Yes, it needs to be sound and rigorous, but its main purpose should be to serve the patients, families and the community. Scientific evaluation of the Insite may have been a necessary condition but it was certainly not a sufficient condition to bring about a permanent change in public policy or a sustainable supervised injection facility.

Sometimes scientists and bureaucrats spend too much time worrying about protecting objectivity at the expense of advocacy. Yet, there are, time and time again, instances where advocacy needs to be undertaken and undertaken vigorously. The supervised injection facility was one of those times. It demanded public advocacy. Despite the victory, on the day of court announcement, I felt relief more than elation. I couldn’t help but wonder what would have happened to Insite if things had been different? If we had lost the court case, would the various stakeholders have chosen the ethically sound course of action by continuing to operate in spite of an unjust law prohibiting the facility’s operation? There would have been a strong ethical case for breaking the law and keeping Insite open. There was no scientific uncertainty about the effectiveness of Insite at engaging a hard to reach population in healthcare and saving lives. The only equipoise was political. Had we lost, would we have been forced to live through a medical, legal and ethical disaster while people died of preventable overdoses because it was against the law? These are dark questions that haunted Insite right up until the very moment that the final decision was rendered. Thankfully, we never had to publically confront what might have been had we lost.

In my view, there are three important cultural ramifications of this case. Firstly, this legal decision says a lot about what it is to be a person, to have personhood, in Canada. Personhood symbolizes our connection to the wider human family [49]. What it is to be a person exists in the borderland of human relations where personal agency and meaning are psychosocially constructed as part of an inner and outer conversation. Socially devalued features of our selves such as addiction reduce our opportunities to be on the threshold of a successful life [10]. The personhood of people with addiction is often undermined or threatened by policies, programs and implicit or explicit exclusion (e.g. drug courts and therapeutic communities are typically founded on the principle that addicts must abdicate a portion of their self-determination). The personhood of people with addictions in Canada has been emphasized and their constitutional rights feature prominently in this legal ruling.

The second consequence of this case is that other jurisdictions may, and should, establish supervised injection if the epidemiological variables demand it. On this point, I disagree with the overemphasis on obtaining consensus from an overly broad collection of stakeholders (e.g. the municipal government, the local police) who are typically consulted in order to obtain their blessing so that lives can be saved by supervised injection. A letter of support from the Chief of Police or Mayor of a city would not be required to establish a cancer treatment program. Correspondingly, one shouldn’t be required in the case of harm reduction programs. If the evidence base is there to support an intervention, then we should move past consensus building when it comes to life saving healthcare. The SCC has ruled that the morality of an activity, such as drug addiction, isn’t enough to ignore someone’s rights to security of their person under section 7 of Canada’s Charter of Rights and Freedoms (the Charter):

“Additionally, the morality of the activity the law regulates is irrelevant at the initial stage of determining whether the law engages a s. 7 right. Finally, the issue of illegal drug use and addiction is a complex one which attracts a variety of social, political, scientific and moral reactions. While it is for the relevant governments to make criminal and health policy, when a policy is translated into law or state action, those laws and actions are subject to scrutiny under the *Charter*” [2] (p.9).

The suitability of supervised injection shouldn’t be debated any longer as though it were on par with a discussion on a sports show about which sports team will win the championship. Supervised injection is healthcare and whether it is required in a jurisdiction needs to be determined by evidence and not arbitrary opinions or fickle political stances in search of votes. If science and medicine have established the best course of action, then we shouldn’t turn to opinion polls to determine the best healthcare (Figure 4).

**Figure 4:**
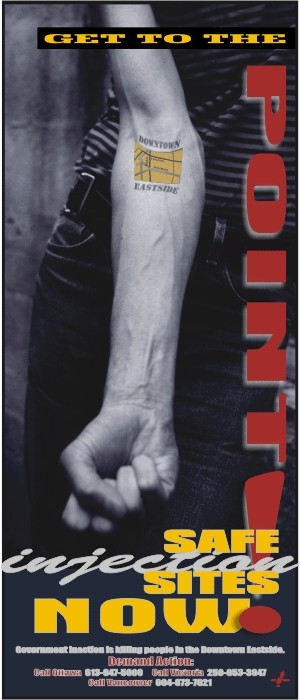
**Safe injection sites now.** Graphic by Flux Design.

Finally, it is my opinion that this ruling provides further affirmation that many healthcare providers know: harm reduction needs to be an explicit part of the standard of care now. Every single health authority and region in Canada should have a proactive policy detailing best practices in harm reduction when it is epidemiologically indicated. It is simply not acceptable to pretend harm reduction doesn’t exist or to let moral opposition rather than evidence based analysis guide decisions in this area.

Any jurisdiction that doesn’t a positive policy on harm reduction is misguided. An example is provided by the City of Abbotsford in British Columbia. In June 2005, Abbotsford amended their zoning (bylaw no. 1378–2004) in order to overtly exclude harm reduction:

“Prohibited uses would include safe injection sites, needle exchanges, mobile dispensing vans, methadone treatment facilities and other types or similar uses.”

The experience of Insite should be an important lesson for jurisdictions that ignore, or in the case of Abbotsford, outlaw, harm reduction as part of healthcare. They do so at their own peril legally, medically and ethically (Figure 5).

**Figure 5:**
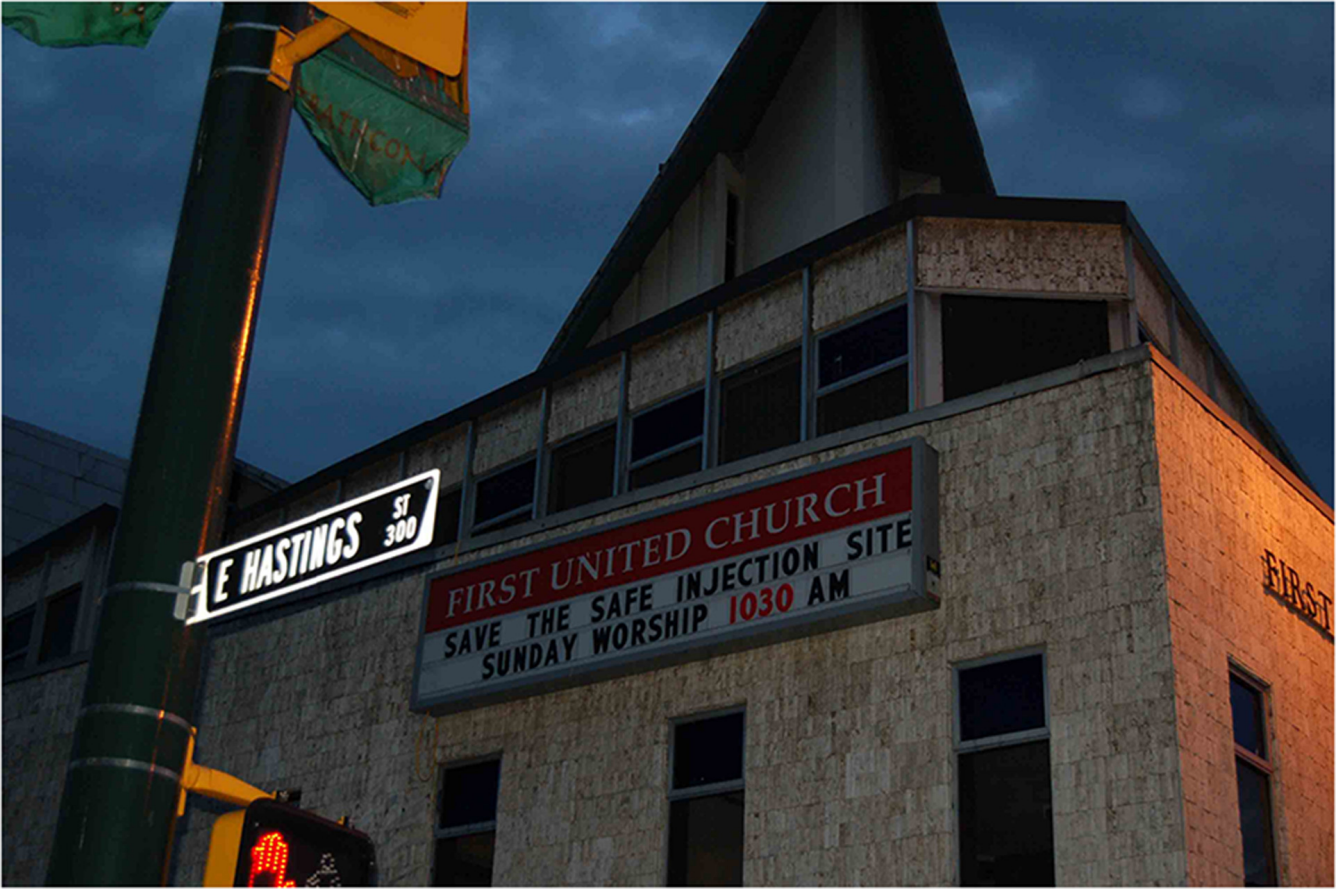
**Church Marquee in Vancouver’s Downtown Eastside.** Photograph by Dan Small.

This commentary is not meant to be a distant scientific paper but, instead, an experience-based and socially positioned interpretation of events that I have lived. As one of the creators of Insite, I had always imagined that establishing an injection site would be the most difficult task to accomplish. In fact, it seems to me that the protection of it, once established, has been an even larger challenge. The struggle to protect Insite has distracted us from many other important prevention, treatment, enforcement and harm reduction initiatives that need to be established to comprehensively address addiction. The fact that the program has survived is itself an indicator of social change and I believe that this ruling signals that we have reached an important cultural milestone. We’ve gone so much further than hoping that addiction will one day be understood as a healthcare matter. The very fact that Insite can exist, with the permanence of a Supreme Court decision, supports harm reduction as part of the standard of care, with the sustainability it deserves. With this ruling, we’ve moved beyond hopefulness to a point in our history where people with addictions have been unchained. Canada’s highest court has spoken. It’s the law.

“If you have built castles in the air, your work need not be lost; that is where they should be. Now put the foundations under them [50] (p.255).

## Competing interests

The author declares that he has no competing interests.
